# E-PTES-S: Enhanced Trust Evaluation via Multidimensional Spatiotemporal Fusion and Variance-Based Stability Sequence Extraction in IoT Sensing Networks

**DOI:** 10.3390/s26082382

**Published:** 2026-04-13

**Authors:** Jinze Liu, Yongtao Yao, Xiao Liu, Jining Chen, Shaoxuan Li, Jiayi Lin

**Affiliations:** 1 School of Computer, Electronics and Information, Guangxi University, Nanning 530004, China; jinze@st.gxu.edu.cn (J.L.); yongtao@st.gxu.edu.cn (Y.Y.); jiayilin@st.gxu.edu.cn (J.L.); 2 Guangxi Key Laboratory of Digital Infrastructure, Guangxi Zhuang Autonomous Region Information Center, Nanning 530201, China; 2112301001@st.gxu.edu.cn; 3 School of Civil Engineering and Architecture, Guangxi University, Nanning 530004, China; shaoxuan@st.gxu.edu.cn

**Keywords:** Internet of Things, trust evaluation, evidence sequence extraction, spatiotemporal evidence fusion, behavioral stability

## Abstract

Mobile data collectors (MDCs) play a very important role in Internet of Things (IoT) sensing networks. However, ensuring their trustworthiness against insider threats, such as on–off attacks and spatiotemporal fabrication, remains a critical challenge. Existing trust evaluation methods frequently struggle with these threats due to insufficient evidence dimensions and the inability to quantify behavioral stability. To address these limitations, this paper proposes an enhanced proactive trust evaluation system based on stability sequence extraction (E-PTES-S). E-PTES-S improves the evaluation accuracy by integrating five factors of evidence, stability-computation mechanisms, and an adaptive weight allocation scheme to maintain robustness even when proactive verification data is scarce. In addition to the usual interaction and proactive verification indicators, regional consistency ($T_{RC}$) and task timeliness ($T_{TT}$) are introduced to mitigate location falsification and transmit-time deviations more rigorously. Then, a sliding window technique is used to obtain an integrated evidence sequence, which includes a new continuous stability sequence ($F_{CSS}$) and traditional credible, untrustworthy, and uncertain sequences. This continuous stability sequence adds a variance-based incentive scheme to measure behavioral stability. Finally, the normalized trust value is derived from multiple indicators including multidimensional spatiotemporal evidence and stability metrics. Experimental results show that the proposed E-PTES-S achieves a normal node detection rate of 98.7% under complex dynamic conditions, outperforming the baseline PTES and Trust-SIoT algorithms by approximately 9% and 1%, respectively, while also improving the cumulative data collection profit by 4.8%. Furthermore, robustness analysis demonstrates that E-PTES-S exhibits excellent robustness against physical-layer uncertainties, successfully sustaining an 84.4% detection rate even under severe environmental shadowing.

## 1. Introduction

Because nodes are the basic units of seamless data creation and exchange in open, heterogeneous, and dynamic environments, their trustworthiness affects the overall security and reliability of an IoT or IoV network [1,2]. In an increasing number of heterogeneous and dynamic environments, IoT devices are widely adopted today [3]. Dynamic trust evaluation refers to updating trust values frequently due to the changing behaviors of IoT devices [4]. Under such circumstances, collaborative willingness and behavioral authenticity of MDCs, acting as a bridge connecting sensing devices and data centres, have become increasingly ambiguous to evaluate. Compromised IoT nodes may use their access privileges for malicious behaviour, such as spoofing identities, launching denial-of-service attacks, and performing lateral movement to disrupt the overall network [5,6]. After the attack is successful, it may cause system failure. Therefore, building a unified and comprehensive node trust assessment system is necessary to ensure the secure operation of the distributed IoT system.

Although traditional network security technologies have effectively resisted external attacks on their static defences, once an attacker gains access to the network under a legitimate identity and operates unnoticed, they will no longer be defended [7,8]. Trust mechanisms can serve as a complement to network security in response to the above problems. According to the behavioral trustworthiness assessment model in this research, the range of the behavioral trustworthiness score is 0 to 1 [9]. The trust evaluation platform monitors the previous interactions of entities to accumulate or decrease their experience scores. If a node shows behavioral consistency (successfully forwarding messages or satisfying truthful information sharing), its trust score rises through reward mechanisms; otherwise, under negative conditions, it experiences a reduction in credibility through penalty mechanisms [10]. Finally, according to the above trust scores, a reliable IoT device is chosen for edge computing applications to avoid introducing a malicious device into the network [11].

At present, various IoT trust evaluation mechanisms have been put forward that employ models including reputation-based trust models, virtual currency trust models and game theory-based models [12]. Among them, trust evaluation systems characterized by proactive probing and sequence extraction (e.g., PTES) have made significant progress. They introduce unmanned aerial vehicles (UAVs) as trusted third parties to collect benchmark data and use sequence-extraction mechanisms to categorize and evaluate the continuous interaction performance of nodes [13,14]. However, when dealing with the increasingly concealed and complex advanced persistent threats originating from within modern IoT environments, existing models reveal two prominent limitations:

First, existing mechanisms inherently rely on logical content verification but lack physical spatiotemporal constraints. Recent security studies emphasize that relying solely on logical verification leaves systems extremely vulnerable to wireless spoofing attacks, necessitating physical-layer signal features for robust spatial authentication [15,16]. Most current trust management approaches (such as PTES) evaluate the general veracity of data and continuous misbehaviors, but are fundamentally blind to spatiotemporal spoofing. Attackers can easily bypass content-focused models by reporting accurate information from falsified locations or deliberately hoarding data to execute stealth delay attacks, even in UAV-assisted networks [17,18,19]. Therefore, our introduced $T_{RC}$ and $T_{TT}$ achieve a transition from purely logical evaluation to physical-spatiotemporal bounded verification, reducing the likelihood of location falsification and data expiration.

Second, existing models are inadequate in quantifying behavioral stability, leaving systems vulnerable to strategic manipulation and malicious behaviors [20]. Sneaky attackers often execute selective misbehaviors (such as on–off and cooperative attacks) by intermittently oscillating between normal and malicious states or colluding with malicious peers to evade threshold-based detection [9,21]. Previous frameworks evaluate nodes based on cumulative averages or basic sequence categories, allowing highly volatile opportunistic nodes to maintain passing scores and evade punishment. The introduction of the variance-based $F_{CSS}$ mechanism addresses this significant flaw.

To overcome the above problems, this paper proposes a multidimensional spatiotemporal evidence fusion-based E-PTES-S model with a continuously extracted stability sequence. Based on the original structure of proactive verification, multiple extension mechanisms have been added to this new system. At the evidence acquisition layer, E-PTES-S introduces regional consistency and task timeliness mechanisms. By utilizing physical signal features such as the received signal strength indicator (RSSI) and timestamps, it forms a 5-D evidence acquisition matrix. This approach effectively overcomes the anti-spoofing limitations of existing systems, providing superior monitoring and prevention capabilities. At the trust computation layer, to measure and reward nodes’ behavioral consistency effectively, we employ a continuous stability sequence (CSS) in the sequence-extraction mechanism. Through the utilization of an exponential penalty-incentive model based on trust variance, it is possible to accurately identify honest nodes and successfully reduce their variance. Malicious nodes can also be detected (with low confidence) if they have high variances but acceptable mean values.

Based on the existing works that build on the PTES framework [14], our research provides new insights or extensions.

- Construction of a Five-Dimensional Trust Evidence Fusion Architecture: We propose an E-PTES-S that seamlessly integrates regional consistency and task timeliness into the original joint active and passive probing model. It has greatly expanded the scope of defending against concealed intrusions by stealth hoarding delay, especially localisation falsification.
- Proposal of a Variance-Based Continuous Stability Sequence Extraction Mechanism: We innovatively define a high-order trust factor, $F_{CSS}$. Unlike conventional approaches based solely on the total probability of success and failing to reveal behaviours, it can ascertain the authority level of nodes through the analysis of behavioral patterns.
- Optimization of an Adaptive Normalization Algorithm: A trust normalisation algorithm and a weight-adaptive allocation scheme are combined in this design. When UAV probing data is insufficient, the system can automatically modify the spatial-level weight of regional consistency during cross-validation to enhance robustness in heterogeneous and resource-limited IoT systems.

## 2. Related Work

This part introduces the research results on IoT trust assessment: multidimensional trust model construction, its deficiencies under a dynamic environment, as well as existing applications of time-series and behaviour-oriented stability analyses in trust evaluation.

### 2.1. Multidimensional Trust Evaluation in the IoT

Trusted assessment has consistently been identified as the primary security safeguard to defend against insiders in IoT sensing networks. In recent years, research has gradually moved away from a general-trust model towards scenario-specific and intelligent systems. Many traditional security mechanisms are not able to work effectively against an attacker whose identity is verified. Therefore, dynamically assessing the trust and credibility of heterogeneous nodes is a pressing demand [22,23].

Multidimensional data is integrated into this work to deal with complex topologies of IoT nodes and evaluate collaboratively. Trust mechanisms have been widely deployed to mitigate malicious insider attacks based on multi-sources of feedback in highly mobile scenarios such as vehicular ad hoc networks (VANETs) and complicated routing situations [24]. In this context, researchers have put forward context-aware trust prediction mechanisms and trust-aware routing schemes for the secure selection of relays and the detection of rogue nodes [24].

In recent years, the integration of UAVs and trust management has become a prominent trend in distributed IoT architectures. To facilitate large-scale and low-power data collection, studies have increasingly utilized UAVs as mobile gateways due to their deployment flexibility and broad coverage [25]. Concurrently, as the scale of IoT and cross-network interactions expands, ensuring the credibility of shared data has driven the development of reputation-driven trust mechanisms to foster secure collaboration [26].

In particular, in UAV-assisted industrial cyber–physical systems, Liu et al. [27] proposed a trust-based active detection (TBAD) scheme to improve the reliability of data collection and reduce data redundancy; for the purpose of improving data scarcity, Huang, M. et al. [14] introduced the PTES system to introduce UAVs as trusted third-party procurers to proactively verify data and provide assurance for trustworthy information collection.

However, the current proactive verification mechanism faces many deficiencies in terms of spatiotemporal dimensions. As highlighted in the studies on trustworthy 6G networks [28] and IoT spatiotemporal data fusion, the new system needs strict spatiotemporal consistency checks to prevent sophisticated location falsification from intruding. Replaying legitimate data from a specific location makes traditional content-focused verification models vulnerable. Based on the current situation, we propose to build an E-PTES-S defence network to achieve both physical $T_{RC}$ and $T_{TT}$.

### 2.2. Time Decay, Sequential Behavior, and Age of Information

Recently, some studies have applied decay functions and adaptable weights to prioritise recent behaviours and reduce the impact of old feedback on the trust evaluation of dynamic malicious devices [29].

Decay alone is insufficient; there will also be other threats such as malicious interference in interactions over an extended period. In an on–off attack, adversaries alternate between benign and malicious behaviors to maintain their trust scores above a detection threshold [8,30]. Recent studies have shown that traditional cumulative mean-based models are highly susceptible to such on–off attacks, necessitating more rigorous penalties such as exponential trust score decay mechanisms [21]. Consecutive packet dropping is frequently a precursor to strategic malicious behavior, as demonstrated by recent hybrid trust models analyzing consecutive delivery failures [31].

Furthermore, optimizing the timeliness of data is essential for the economic viability of crowdsensing systems. Recently, alongside strict latency optimizations in edge-enabled networks [32], age of information (AoI) has emerged as an end-to-end metric to characterize latency in status updating systems [33]. Advanced scheduling policies and resource allocation schemes have been proposed to minimize AoI and guarantee real-time reliability in vehicular and UAV-assisted mobile crowdsensing networks [34,35]. This provides strong theoretical validation for incorporating the task timeliness mechanism in our work, which identifies and selects high-quality nodes that provide the freshest data.

While existing works have made significant progress in multidimensional trust evaluation and time-window analysis, there remains a distinct lack of a unified framework that simultaneously addresses spatiotemporal spoofing and deeply quantifies behavioral stability. Existing sequence extraction methods fail to differentiate between consistently honest nodes and highly volatile opportunistic nodes that manage to maintain passing average scores. To narrow this gap, our E-PTES-S framework introduces CSS; it uses a variance-based incentive scheme to penalise trust oscillations and reward long-term stability while rigorously enforcing data freshness and spatial authenticity.

## 3. System Model

### 3.1. Network Entities and Hierarchical Architecture

**Figure 1:**
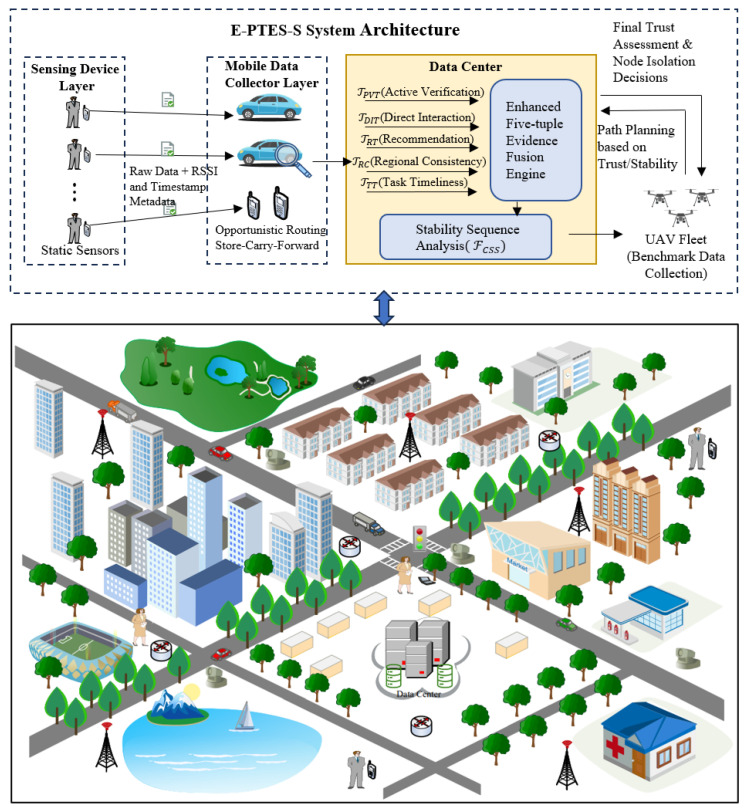
Network model of E-PTES-S.

This study builds a typical hierarchical IoT urban-sensing data-collecting system. According to Figure 1, there are three essential parts of the network architecture: a lower-level sensor node system; an intermediate wireless data collection module; and an upper-level application processing and information management platform.

Sensing device layer: This layer is composed of *k* static sensing devices spread in target monitoring areas, such as urban main streets or environmental monitoring points. As reliable data sources in the system, these devices generate raw sensing data for use by other parts of the system. They are also functionally enhanced to support the regional consistency verification mechanism proposed by E-PTES-S. Sensing devices are set up to record the physical layer signals (such as RSSI) and accurate interaction timestamps between them and mobile nodes; afterwards, these records will be encrypted and packaged together with the sensed information. This mechanism guarantees that the physical location and time attribute information of the data are not tampered with later, providing a basis for verifying its originality.

Mobile data collector layer: This intermediate layer has *N* intelligent terminals with mobility that function as MDCs. Primarily, there are two categories: smart cars with onboard communication equipment and portable intelligent terminals. As the critical bridge connecting static sensors to a cloud data centre, MDCs travel on fixed paths or temporary routes; they establish short-distance links with sensing devices in their transmission area through opportunistic route switching for store–carry–forward services. At this layer with its openness and mobility, MDCs are likely to be more prone to attacks than other network nodes; thus, they need to be verified based on trustworthiness. According to the E-PTES-S architecture in this study, besides evaluating whether the MDC forwards its own traffic normally, it will be judged based on the accuracy of transmitting location information and timely transmission of completed tasks as well. Malicious MDCs use techniques such as location falsification, task reward diversion for targeted areas, and delayed data submission; they can also conduct on–off deception by switching from clean to dirty states repeatedly. Therefore, in this system, MDCs are regarded as unreliable nodes that need to be verified continuously and repeatedly by the data centre.

Data processing and management layer: This layer consists of a data center (DC) equipped with robust computational and storage capabilities, serving as the decision-making core of the entire architecture.

Unlike traditional models that merely receive data passively, this layer deploys an enhanced five-dimensional evidence fusion engine. It is responsible for calculating a five-dimensional trust vector, which includes proactive verification trust ($T_{PVT}$), direct interaction trust ($T_{DIT}$), recommendation trust ($T_{RT}$), $T_{RC}$ and $T_{TT}$. The DC dynamically manages a fleet of UAVs. Guided by current trust evaluation results and the $F_{CSS}$ analysis, the DC plans UAV flight paths to target regions with questionable trust scores or high data popularity to collect benchmark data, thereby verifying the authenticity of content submitted by MDCs. Ultimately, based on the final normalized trust values, the DC isolates malicious nodes and discards their submitted spatiotemporally anomalous data, consequently maximizing data collection profitability and security.

### 3.2. Variance-Based Stability Incentive Strategy

To defend against opportunistic adversaries such as on–off attackers who manipulate their average trust scores, the system integrates a variance-based stability incentive model. Traditional cumulative models fail to distinguish between consistently honest MDCs and strategic attackers who intermittently oscillate between benign and malicious states. Therefore, the DC monitors the variance of continuous credible interactions. Instead of applying a universal penalty to all fluctuating behaviors, the system filters sequences using a strict stability threshold.

For CSSs that meet this low-variance condition, the system applies an exponential incentive mechanism, defined by the core term $e^{-\mathrm{Var}}$, to rapidly elevate their final normalized trust. When the behavior variance approaches zero, this incentive factor maximizes, heavily rewarding consistently stable nodes. As variance increases within the allowable threshold, the exponential decay naturally diminishes the reward advantage. This strategic model guarantees that long-term behavioral consistency becomes a prerequisite for achieving elite trustworthiness in the network.

## 4. Proposed E-PTES-S System

### 4.1. Overview of E-PTES-S

**Figure 2:**
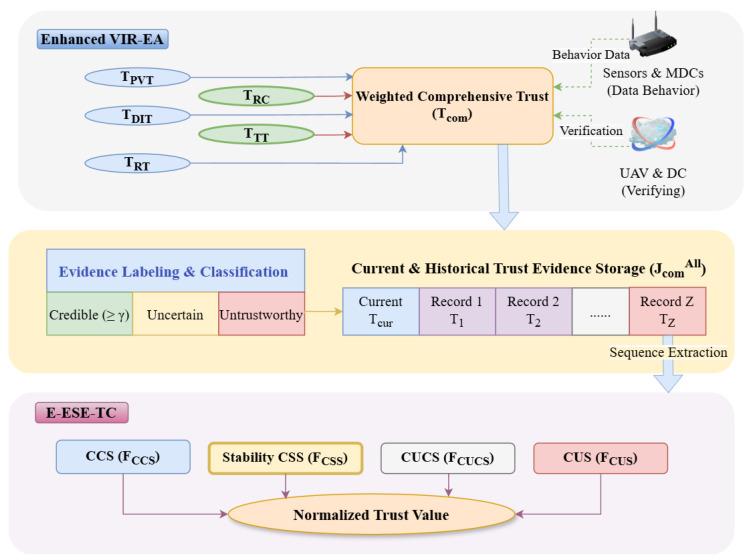
Overall architectural network model of E-PTES-S.

This section elaborates on the proposed E-PTES-S. Functionally and architecturally, this study establishes a direct evolutionary relationship with the proactive trust evaluation system (PTES) proposed by Huang et al. [14]. In terms of similarities, our E-PTES-S inherits the core baseline methodologies of PTES: both frameworks target dynamic IoT crowdsensing networks; both utilize UAVs as trusted third-party procurers to conduct proactive benchmark verification; and both adopt a time-based sliding window mechanism alongside a sequence-extraction concept to store and categorize historical interaction records. As illustrated in Figure 2, building upon this foundational architecture, E-PTES-S further introduces two novel evidence dimensions and a stability-aware sequence-extraction mechanism to address the inherent spatiotemporal limitations of existing approaches. The fundamental rationale of E-PTES-S is that a comprehensive trust evaluation of MDCs must consider not only the data they provide (content correctness) and their communication methods (interaction reliability) but also their claimed locations (spatial authenticity), data delivery timing (temporal timeliness), and behavioral consistency (behavioral stability). Five-faceted views help identify hidden threats that have yet to be discovered through the three-dimensional trust method [14].

According to the modules shown in Figure 2, there are five types of trust evidence: $T_{PVT}$ derived from UAV benchmark data acquisition; $T_{DIT}$, which assesses the communication success rate among MDCs; $T_{RT}$ offered by authoritative entities; the newly proposed $T_{RC}$ technology for identifying location spoofing using physical signals as features; and the newly added $T_{TT}$ assessment of data freshness. By fusing the five evidence types using a weighted method, a comprehensive trust index $T_{com}$ is generated, which is then classified into three categories: credibility, uncertainty, or unreliability based on threshold α and γ.

Trust evidence storage uses a sliding window technique to store the last *Z* pieces of trust information for an MDC, maintaining a temporal distribution of behavioural data. The above time arrangement is essential for following subsequences. The enhanced ESE-TC module is the primary innovative part of this system. In addition to the conventional continuous credible sequence (CCS), continuous untrustworthy sequence (CUS), and continuous uncertain sequence (CUCS), we also propose a fourth novel sequence type, namely the CSS. As a high-order subsequence extracted from the CCS, the CSS has little trust value variation and can represent MDCs with highly stable and dependable behaviour. The four types of sequences have different trust influence values: ($F_{CCS}$, $F_{CUS}$, $F_{CUCS}$, and $F_{CSS}$) according to the length, freshness, and behaviour deviation of interactions. Finally, the full-aggregation function incorporates all four of these elements into one for normalisation while adding stability as a separate factor in proportion and form.

The workflow of E-PTES-S begins with the five-dimensional behavioral monitoring of MDCs. Initially, the system calculates $T_{PVT}$ by comparing MDC-submitted data with benchmark data collected by UAVs. Concurrently, it tracks the number of successful and failed communications to derive the direct interaction trust and, when necessary, acquires recommendation trust through credible recommendation chains. Building on this, the newly added regional consistency trust evaluates spatial authenticity by matching RSSI signals to GPS locations. In contrast, the task timeliness trust measures data freshness based on the time difference between data generation and upload. Upon acquiring all five evidence types, the system fuses them into a $T_{com}$ using adaptive weights, and categorizes it as credible, uncertain, or untrustworthy based on the α and γ thresholds. These labeled pieces of evidence are then stored in a sliding window matrix, retaining the most recent *Z* records. During the trust computation phase, the system first extracts the CCS, CUS, and CUCS from storage, then filters the CSS from the CCS based on low-variance conditions. Then it calculates the trust-influence scores of each sequence form. The CCS and CUS keep products in the form of decaying time and sequence length; however, the CUCS uses a similar expression without penalty. The CSS enhances the stabiliser, which includes both exponential variance terms and superlinear lengths of rewards. Finally, all of the influencing factors are substituted into a more refined normalisation formula to derive the MDC’s trust score.

### 4.2. Main Symbols

Table 1 presents the symbol table of this article to summarise the main mathematical symbols used in the system, define them, and state any associated definitions or equations.

### 4.3. Multimodal Evidence Acquisition Mechanism

This mechanism introduces two novel trust dimensions to the original three evidence sources, thereby expanding the evidence acquisition framework into a comprehensive five-dimensional model. This five-dimensional approach thoroughly covers potential attack vectors and enables robust cross-validation across diverse behavioral aspects.

$T_{PVT}$ verifies the existence of anomalous data behaviors in the evaluated MDC by comparing the data submitted by the MDC with the benchmark data collected by UAVs. For each data packet $D_{x}=\left[t_{c},Site_{c},Dev_{c},Data\right]$, its data quality is represented by *U*-dimensional attributes. Let $Q_{D_{x}}^{i}$ denote the quality of the *i*-th attribute and $\omega_{i}$ denote the attribute weight. The normalized data quality is expressed as:

(1) $$Q_{D_{x}}=\sum_{i=1}^{U}\omega_{i}Q_{D_{x}}^{i},\;\sum_{i=1}^{U}\omega_{i}=1,\;0\leq \omega_{i}\leq 1.$$

Let the benchmark dataset collected by UAVs be $D_{BASE}=\left\{D_{BASE}^{1},D_{BASE}^{2},\ldots \right\}$. For the data $D_{x}$ submitted by the MDC, its corresponding benchmark data is denoted as $D_{BASE}^{x}$. If the data quality difference $|Q_{BASE}^{D_{x}}-Q_{D_{x}}|$ is less than the threshold $\theta_{d}$, it is recorded as a successful data interaction ($C_{s,d}=1$, $C_{f,d}=0$); otherwise, it is classified as a failure ($C_{s,d}=0$, $C_{f,d}=1$):

(2) $$C_{s,d}=\left\{\begin{array}{cc} 1, & \mathrm{if}\;\sum_{i=1}^{U}\omega_{i}\left|Q_{BASE,D_{x}}^{i}-Q_{D_{x}}^{i}\right|\leq \theta_{d},\\ 0, & \mathrm{else}, \end{array}\right.$$

(3) $$C_{f,d}=\left\{\begin{array}{cc} 1, & \mathrm{if}\;\sum_{i=1}^{U}\omega_{i}\left|Q_{BASE,D_{x}}^{i}-Q_{D_{x}}^{i}\right|>\theta_{d},\\ 0, & \mathrm{else}. \end{array}\right.$$

Based on the cumulative number of successes $c_{s}=\sum c_{s,d}$ and failures $c_{f}=\sum c_{f,d}$, the proactive verification trust is calculated using a Bayesian expected smoothing formulation:

(4) $$T_{PVT}=\frac{c_{s}}{c_{s}+c_{f}+1}+\frac{1}{2}\cdot \frac{1}{c_{s}+c_{f}+1}.$$

Direct interaction trust ($T_{DIT}$) is obtained by accumulating the number of successful communications $c_{s,c}$ and failed communications $c_{f,c}$, and subsequently substituting them into the equation where $c_{s}=\sum c_{s,c}$ and $c_{f}=\sum c_{f,c}$. When two MDCs lack historical interaction, third-party recommendation trust ($T_{RT}$) must be introduced. Assuming the recommendation path traverses $L_{rec}$ intermediate recommenders, the direct trust of the ultimate recommender toward the target is $J_{obj}$, and the trust degree of each node on the path toward the next hop is $R_{i,i+1}$, the recommendation trust is calculated as follows:

(5) $$T_{RT}=J_{obj}\prod_{i=1}^{L_{rec}}R_{i,i+1}.$$

When multiple recommendations are received, the system prioritizes the one with the minimum recommendation tier; if the tiers are identical, the recommendation from the most trusted recommender is selected.

Regional consistency trust is a novel evidence dimension introduced to combat location spoofing attacks. This mechanism utilizes the physical propagation characteristics of wireless signals and the geometric constraints of GPS information to construct a collaborative spatial verification filter. To prevent sophisticated attackers from manipulating transmit power to forge RSSI, E-PTES-S introduces a multi-node collaborative verification mechanism. When a target MDC reports its data, a set of nearby witnessing nodes, denoted as W (including adjacent MDCs, static sensors, and active UAVs), simultaneously captures its RSSI. According to the log-distance path loss model, expressed as $RSSI=RSSI_{0}-10n\log_{10}\left(d_{est}/d_{0}\right)+X_{\sigma }$, the estimated distance $d_{est,j}$ between the target and the *j*-th witnessing node can be derived. Here, $RSSI_{0}$ is the reference signal strength at distance $d_{0}$, *n* is the path loss exponent, and $X_{\sigma }$ represents Gaussian shadow fading. In our urban vehicular sensing scenario, *n* is empirically set between 2.7 and 3.5 to reflect typical urban canyon environments. While RSSI is inherently sensitive to multipath fading, our design mitigates this limitation by relying on a multi-node collaborative verification (W witnesses); aggregating independent RSSI readings significantly reduces the variance caused by localized environmental noise. Concurrently, the claimed distance $d_{claim,j}$ is calculated using their reported GPS coordinates. The spatial deviation for the *j*-th witness is defined as $\Delta d_{j}=\left|d_{est,j}-d_{claim,j}\right|$.

The collaborative regional consistency evaluation function for a single interaction is defined by aggregating the verifications from all |W| witnesses:

(6) $$E_{RC}=\frac{1}{\left|W\right|}\sum_{j\in W}\left\{\begin{array}{cc} 1, & \mathrm{if}\;\Delta d_{j}\leq \delta_{th}\\ e^{-\lambda_{loc}\left(\Delta d_{j}-\delta_{th}\right)}, & \mathrm{if}\;\Delta d_{j}>\delta_{th} \end{array}\right.$$

where $\delta_{th}$ is the tolerance threshold and $\lambda_{loc}$ is the penalty coefficient. Within an evaluation period, if an MDC engages in *M* interactions, its regional consistency trust is given by:

(7) $$T_{RC}=\frac{\sum_{k=1}^{M}E_{RC}^{\left(k\right)}}{M}.$$

By relying on multiple independent witnesses rather than a single point of failure, this multi-node verification strictly quantifies spatial consistency, significantly enhancing the space protection ability against localized spoofing.

To systematically analyze the error propagation caused by environmental dynamics (shadowing and multipath) and node heterogeneity, we quantify the distance estimation variance. Let the total RSSI measurement noise be modeled as $X_{\sigma }∼N\left(0,\sigma_{env}^{2}+\sigma_{het}^{2}\right)$, where $\sigma_{env}^{2}$ represents the environmental shadowing variance and $\sigma_{het}^{2}$ denotes the variance introduced by hardware heterogeneity. Using a first-order Taylor series expansion on the log-distance path loss model, the error propagation to the estimated distance $d_{est}$ can be approximated as:

(8) $$\mathrm{Var}\left(d_{est}\right)\approx {\left(\frac{\partial d_{est}}{\partial RSSI}\right)}^{2}\mathrm{Var}\left(X_{\sigma }\right)={\left(\frac{d_{est}\ln10}{10n}\right)}^{2}\left(\sigma_{env}^{2}+\sigma_{het}^{2}\right)$$

This formulation reveals that the distance estimation error scales proportionally with the physical distance and the combined noise variance, confirming that individual RSSI measurements are highly sensitive in complex real-world scenarios. However, E-PTES-S mitigates this inherent vulnerability through the multi-node collaborative verification mechanism. Assuming that shadowing effects are approximately independent for sufficiently separated witnesses, aggregating the spatial deviations from |W| nodes effectively suppresses the overall error variance:

(9) $$\mathrm{Var}\left(\bar{\Delta d}\right)=\frac{1}{{\left|W\right|}^{2}}\sum_{j\in W}\mathrm{Var}\left(d_{est,j}\right)\approx \frac{\mathrm{Var}(d_{est})}{\left|W\right|}$$

This inverse mathematical relationship (∝1/|W|) rigorously demonstrates that as the number of independent witnesses increases, the adverse effects of multipath fading, shadowing, and node heterogeneity are statistically smoothed out. Furthermore, in edge cases where an MDC travels to sparse areas with insufficient witnesses, such as when |W|≤1, the system relies on the temporal continuity of $F_{CCS}$ and the remaining evidence dimensions to compensate for the temporary drop in spatial confidence, thereby preventing an abrupt and unjustified penalty. Consequently, this multi-dimensional cross-validation guarantees the robustness of $T_{RC}$ under complex and dynamic wireless conditions.

Task timeliness trust introduces another vital defense against data freshness intruders, such as stealth hoarding and delay attacks. To strictly quantify timeliness, the system extracts the data generation timestamp $t_{gen}$ signed by the static sensor and the upload timestamp $t_{up}$ recorded by the DC upon reception. Assuming global clock synchronization via the Network Time Protocol (NTP) with an allowable drift margin, the absolute transmission delay is calculated as $\tau_{x}=t_{up}-t_{gen}$ (in milliseconds).

The evaluation defines a “timely task” based on an optimal delay threshold $\tau_{opt}$, which accounts for normal queuing, processing, and propagation delays inherent to the specific sensing task. To address the practical challenge of achieving strict millisecond-level global clock synchronization in heterogeneous IoT environments, the inevitable clock drift and network jitter are explicitly absorbed into the design of $\tau_{opt}$. By configuring $\tau_{opt}$ and $\tau_{max}$ at a macro-level scale of seconds or minutes, the system inherently tolerates micro-level synchronization errors, ensuring that benign nodes are not falsely penalized for minor delays. The maximum tolerable delay is set at $\tau_{max}$. The timeliness utility function for a single task calculates the deviation penalty:

(10) $$U_{time}\left(\tau_{x}\right)=\left\{\begin{array}{cc} 1, & \mathrm{if}\;\tau_{x}\leq \tau_{opt}\\ \frac{\tau_{max}-\tau_{x}}{\tau_{max}-\tau_{opt}}, & \mathrm{if}\;\tau_{opt}<\tau_{x}\leq \tau_{max}\\ 0, & \mathrm{if}\;\tau_{x}>\tau_{max} \end{array}\right.$$

Among $N_{data}$ pieces of information uploaded by an MDC within the evaluation period, $T_{TT}$ is aggregated as a priority-weighted average:

(11) $$T_{TT}=\frac{\sum_{x=1}^{N_{data}}w_{pri}^{\left(x\right)}\cdot U_{time}\left(\tau_{x}\right)}{\sum_{x=1}^{N_{data}}w_{pri}^{\left(x\right)}}$$

where $w_{pri}^{\left(x\right)}$ stands for the priority weight of the specific data packet. $T_{TT}$ directly penalizes delayed submissions. Ultimately, as defined in Equation (12), $T_{TT}$ and $T_{RC}$ are adaptively weighted and fused with the other three dimensions to generate $T_{com}$, which is then categorized and appended into $T_{com}^{All}$ as an integrated multidimensional evidence record.

After obtaining the trust evidence of all kinds, they should be integrated and formed as $T_{com}$. We employ an adaptive weighted fusion formula:

(12) $$T_{com}=\omega_{p}T_{PVT}+\omega_{d}T_{DIT}+\omega_{r}T_{RT}+\omega_{rc}T_{RC}+\omega_{tt}T_{TT}$$

where the weight vector $\left[\omega_{p},\omega_{d},\omega_{r},\omega_{rc},\omega_{tt}\right]$ satisfies the normalization condition ∑ω=1. To adaptively adjust the spatial level weights when UAV probing data is insufficient, we introduce a UAV data availability ratio $\rho_{uav}=\min\left(1,\frac{N_{uav}}{Th_{uav}}\right)$, where $N_{uav}$ is the number of benchmark data packets collected by UAVs within the current sliding window, and $Th_{uav}$ is the predefined minimum threshold for sufficient verification.

The adaptive heuristic rules for the weight allocation are defined as follows:

(13) $$\left\{\begin{array}{c} \omega_{p}=\rho_{uav}\cdot \omega_{p}^{max}\\ \omega_{rc}=\omega_{rc}^{base}+\zeta \cdot \left(1-\rho_{uav}\right)\\ \omega_{tt}=\omega_{tt}^{base}+\xi \cdot \left(1-\rho_{uav}\right) \end{array}\right.$$

where $\omega_{p}^{max}$ is the maximum weight assigned to $T_{PVT}$ when UAV data is fully sufficient ($\rho_{uav}=1$). $\omega_{rc}^{base}$ and $\omega_{tt}^{base}$ are the foundational weights for $T_{RC}$ and $T_{TT}$, respectively. ζ and ξ are compensatory coefficients that proportionally increase the system’s reliance on regional consistency and task timeliness when $\rho_{uav}<1$. To ensure system stability, these coefficients are quantitatively bounded to prevent the system from over-relying on purely physical constraints. Specifically, the adjusted weights satisfy $\omega_{rc}^{base}+\zeta \leq 0.35$ and $\omega_{tt}^{base}+\xi \leq 0.30$. This constraint ensures that even when UAV data is completely unavailable ($\rho_{uav}=0$), the combined weight of direct interaction trust and recommendation trust remains at least 0.35, thereby preserving the fundamental interaction-based trust evaluation capability.

Consequently, the remaining weights, $\omega_{d}$ and $\omega_{r}$, are proportionally scaled based on their foundational weights to ensure the normalization condition ∑ω=1 is satisfied. The specific scaling formulas are defined as:

(14) $$\omega_{d}=\omega_{d}^{base}\cdot \frac{1-(\omega_{p}+\omega_{rc}+\omega_{tt})}{\omega_{d}^{base}+\omega_{r}^{base}}$$

(15) $$\omega_{r}=\omega_{r}^{base}\cdot \frac{1-(\omega_{p}+\omega_{rc}+\omega_{tt})}{\omega_{d}^{base}+\omega_{r}^{base}}$$

Given that $\omega_{d}^{base}+\omega_{r}^{base}>0$, this adaptive strategy ensures robust five-dimensional fusion: when proactive verification is limited, the system automatically strengthens the spatiotemporal physical constraints ($T_{RC}$ and $T_{TT}$) to prevent location spoofing and delay attacks, maintaining high evaluation accuracy in resource-constrained IoT scenarios.

### 4.4. Trust Evidence Storage Mechanism

After obtaining the overall five-dimensional trust, $T_{com}$, the system enters the evidence storage stage. Initially, two thresholds, α and γ (0≤α≤γ≤1), are introduced to discretize and label $T_{com}$. Specifically, when $0\leq T_{com}<\alpha$, the evidence is labeled as untrustworthy; when $\alpha \leq T_{com}<\gamma$ it is labeled as uncertain; and when $\gamma \leq T_{com}\leq 1$, it is labeled as credible. Each evidence record encapsulates the MDC identifier, the comprehensive trust value, and the corresponding category label. Subsequently, a temporal sliding window mechanism is employed to retain the most recent *Z* evidence records. As new evidence arrives, preceding records are sequentially shifted backward, and any records exceeding the window capacity are systematically discarded. For a specific evaluated MDC *a*, its trust evidence sequence is stored as $\left\{T_{cur},T_{com}^{Z},T_{com}^{Z-1},\ldots ,T_{com}^{1}\right\}$, and the evidence corresponding to all *N* MDCs constitutes the following storage matrix:

(16) $$T_{com}^{All}=\left[\begin{array}{cccc} T_{cur}^{1} & T_{com}^{1,Z} & \ldots & T_{com}^{1,1}\\ T_{cur}^{2} & T_{com}^{2,Z} & \ldots & T_{com}^{2,1}\\ \vdots & \vdots & \ddots & \vdots \\ T_{cur}^{N} & T_{com}^{N,Z} & \ldots & T_{com}^{N,1} \end{array}\right].$$

The order of this time should serve to retrieve continuous sets of information and capture behavioural characteristics.

### 4.5. Enhanced Evidence Sequence Extraction-Based Trust Computation Mechanism

The E-ESE-TC mechanism is the main theoretical innovation proposed in this paper. We classify marginally qualified nodes by using the CSS as the fourth category compared with exceptionally steady and dependable nodes. To accurately capture multiple stable behavioral phases without redundant calculations, the system evaluates all contiguous sub-segments within the CCS. A candidate qualifies as a CSS subject to two conditions: all its elements must be credible, and the variance of its trust values, denoted as $\mathrm{Var}\left(S\right)=\frac{1}{L}\sum_{j=1}^{L}{\left(T_{t_{j}}-\bar{T}\right)}^{2}$, must not exceed the stability threshold $\delta_{stb}$. Subsequently, the system filters these candidates to retain only the maximal non-overlapping sequences. The CSS is responsible for identifying elite MDCs that are both credible and have relatively stable behaviours to meet the conditions for adding more trust rewards.

With the introduction of the CSS, the final trust score needs to consist of four parts: a positive trust part calculated from the CCS, a negative trust component resulting from the CUS, a propensity-trust element derived from the CUCS, and an improvement-factor for stability provided by the CSS. We present a modified normalised trust function:

(17) $$Tru_{i}=\frac{F_{CCS}+F_{CSS}}{\chi_{T}}+\frac{F_{CCS}+F_{CSS}}{\left(F_{CCS}+F_{CSS}+F_{CUS}\right)}\cdot \frac{F_{CUCS}}{\chi_{T}}$$

where the normalization factor is $\chi_{T}=F_{CCS}+F_{CUS}+F_{CUCS}+F_{CSS}$. To prevent division-by-zero errors and facilitate cold starts for newly joined MDCs lacking historical evidence, the system assigns a default initial trust value. Additionally, to avert localized singularity when a node possesses only uncertain sequences, a sufficiently small positive constant ε is implicitly added to the denominator $\left(F_{CCS}+F_{CSS}+F_{CUS}\right)$. The above-mentioned equation reveals that enhanced stability credibility behaviours are directly responsible for trustworthiness; when dealing with uncertainty, assigning greater weight to highly stable nodes preserves the system’s overall differentiating capability.

The computation of the positive trust value, $F_{CCS}$, considers both time decay and sequence length:

(18) $$F_{CCS}=\sum_{i=1}^{\kappa }\beta^{t-t_{i,near}}\cdot L_{i}^{CCS}$$

where κ is the total number of CCSs; β represents a time-decay factor (0<β<1), $t-t_{i,near}$ refers to the time interval from the most recent contact in this sequence until now; and $L_{i}^{CCS}$ stands for the length of the *i*-th sequence. The inclusion of elements such as regional consistency and timeliness makes it harder to reach long $F_{CCS}$. Therefore, $F_{CCS}$ becomes a more precise indicator of genuine excellent results.

The negative trust value, $F_{CUS}$, implements strict punishment measures by using an exponential penalty for continuous untrustworthy behaviour:

(19) $$F_{CUS}=\sum_{i=1}^{\eta }\frac{L_{i}^{CUS}}{\beta^{t-t_{i,far}}}\cdot \frac{\sigma \tan[(\kappa +1)/T-\sigma ]+\sigma \tan\sigma }{\pi /2+\sigma \tan\sigma }$$

where η is the total number of CUSs, $t-t_{i,far}$ represents the time difference between the earliest interaction of the sequence and the current time, $L_{i}^{CUS}$ is the sequence length, and σ controls the penalty intensity. The term (κ+1)/T reflects the proportion of credible sequences, where *T* denotes the total number of all extracted sequences. Within the framework, this penalty is aimed at both content tampering and persistent location spoofing and delay attack acts; therefore, it provides a complete deterrent for malfeasance.

The factor $F_{CSS}$ exerts a significant impact on the final evaluation.

(20) $$F_{CSS}=\sum_{j=1}^{M}\beta^{t-t_{j,near}}\cdot {\left(L_{j}^{CSS}\right)}^{\lambda }\cdot e^{-\mathrm{Var}(S_{j}^{CSS})}$$

where *M* is the total number of CSSs, $\beta^{t-t_{j,near}}$ represents the time decay, $L_{j}^{CSS}$ is the sequence length, λ>1 serves as the length reward exponential with super-linear rewards, $\mathrm{Var}(S_{j}^{CSS})$ indicates the sequence variance, and $e^{-\mathrm{Var}(S_{j}^{CSS})}$ acts as the core stability term. As the variance approaches zero, this term equals 1; otherwise, it decreases sharply due to increased variation, thereby distinguishing true good performance nodes from unstable ones more precisely.

For continuous uncertain sequences, the trust influence value is obtained through calculation:

(21) $$F_{CUCS}=\sum_{i=1}^{\mu }\frac{L_{i}^{CUCS}}{\beta^{t-t_{i,far}}}$$

where μ denotes the total number of CUCSs.

### 4.6. Algorithm Pseudocode

Algorithm 1 summarises the main process for evaluating trust based on the E-PTES-S approach: evidence acquisition, storage and trust calculation.
**Algorithm 1** E-PTES-S Trust Evaluation Algorithm
**Require:** $D_{int}$, $D_{BASE}$, $L_{GPS}$, Θ {thresholds and parameters}**Ensure:** Tru {array of normalized trust values} Phase 1 & 2: Evidence Acquisition and Storage  1:**for** each $MDC_{i}\in M$ **do**  2:    Compute $T_{PVT},T_{DIT},T_{RT},T_{RC},T_{TT}$ via respective spatiotemporal models  3:    $T_{com}\leftarrow \omega_{p}T_{PVT}+\omega_{d}T_{DIT}+\omega_{r}T_{RT}+\omega_{rc}T_{RC}+\omega_{tt}T_{TT}$  4:    $label\leftarrow \mathrm{classifyEvidence}(T_{com},\alpha ,\gamma )$  5:    $T_{com}^{All}\left[i\right]\leftarrow \mathrm{appendAndSlide}\left(\left(T_{com},label\right),Z\right)$  6:**end for**** ** Phase 3: Sequence Extraction and Trust Computation  7:**for** each $MDC_{i}\in M$ **do**  8:    $S_{CCS},S_{CUS},S_{CUCS}\leftarrow \mathrm{extractBaseSequences}\left(T_{com}^{All}\left[i\right]\right)$  9:    $S_{CSS}\leftarrow \emptyset$10:    **for** each contiguous subsequence sub of $S_{CCS}$ **do**▷ Ensure continuity11:        **if** $\mathrm{Var}\left(sub\right)\leq \delta_{stb}$ **then**12:           $S_{CSS}\leftarrow S_{CSS}\cup \left\{sub\right\}$▷ Extract stable subsequences13:        **end if**14:    **end for**15:    $S_{CSS}\leftarrow \mathrm{getMaximalNonOverlapping}\left(S_{CSS}\right)$▷ Filter redundant subsets16:    Compute $F_{CCS},F_{CUS},F_{CUCS}$ utilizing time decay functions17:    $F_{CSS}\leftarrow \mathrm{calStabilityReward}\left(S_{CSS},\beta ,\lambda \right)$▷ Apply super-linear reward18:    $\mathrm{Tru}\left[i\right]\leftarrow \mathrm{normalizeTrust}\left(F_{CCS},F_{CSS},F_{CUS},F_{CUCS}\right)$19:**end for**20:**return** Tru

## 5. Experiment and Performance Analysis

This part evaluates the effectiveness of this paper’s E-PTES-S scheme in a series of simulations. Under the same experimental condition, we compare the E-PTES-S framework with a baseline PTES scheme [14] and another Trust-SIoT system [36] to assess its overall performance from different perspectives in detail.

### 5.1. Experimental Setup

As the data source for this experiment, the T-Drive taxi trajectory dataset [37,38] provided by Microsoft Research Asia contains a large number of taxi GPS coordinates in Beijing over more than seven days, which cover most of the main urban roads and truly record the mobility behaviour of MDCs within cities. A portion of the taxi movements in the above-mentioned dataset is selected to form a mobile crowd-sensing system; taxis serve as MDCs, while multiple such stations are placed within the range they operate.

In the simulation, the attack forms of malicious nodes include four kinds: collusion attacks, data tampering, on–off attacks, and bad-mouth/good-mouth attacks; they are all randomised and evenly divided between the attacker group. The data center dispatches UAVs to collect benchmark data at hotspot stations to verify the reliability of data submitted by MDCs proactively. During each data collection round, MDCs navigate along their empirical trajectories, interact with sensors within their communication range to submit sensing data, and simultaneously communicate with other nearby MDCs. Comparative experiments are conducted across 10 distinct rounds of data collection against two baseline schemes to ensure a comprehensive performance evaluation: the PTES framework and the Trust-SIoT framework.

PTES Framework [14]: This serves as the primary baseline. It utilizes UAVs as trusted third parties for proactive verification and employs a basic sequence-extraction mechanism (classifying behaviors into credible, untrustworthy, and uncertain sequences) without considering deep spatiotemporal constraints or continuous behavioral variance.

Trust-SIoT Framework [36]: To demonstrate the superiority of E-PTES-S against modern machine-learning-driven approaches, we adapted the Trust-SIoT model as the second baseline. Originally designed for the Social Internet of Things, Trust-SIoT employs an artificial neural network (ANN) to classify trustworthy objects by capturing complex non-linear relationships. It integrates five trust metrics: direct trust, reliability, benevolence, recommendations, and the degree of relationships evaluated via knowledge graph embedding (KGE). To adapt this framework to our mobile crowdsensing scenario, the social relationship metrics of Trust-SIoT (e.g., co-location and co-work object relationships) were directly mapped to the physical interactions and spatial proximities among the MDCs in the T-Drive dataset.

The simulation experiments in this study were developed and executed in a python environment on a computing platform equipped with an Intel Core i7-14700HX processor. To simulate the mobility of nodes in mobile crowdsensing networks, we employed the T-Drive dataset provided by Microsoft Research Asia, which contains real taxi trajectories in Beijing. Within a metropolitan area defined by longitude [115.5,117.5] and latitude [39.4,41.0], K=500 static sensing devices were randomly deployed. A total of N=1600 taxis were selected as MDCs, with the effective communication radius between an MDC and a sensing device set to 50 m. To rigorously evaluate the robustness of the model under complex threats, 10% of the MDCs (160 nodes) were designated as malicious entities, which randomly perform collusion, data tampering, on–off attacks, and bad/good-mouth attacks. The simulation process consisted of 10 data collection rounds.

Regarding the core parameter configuration for trust evaluation, the sliding window size for evidence (*Z*) was set to 10. The predefined minimum threshold for sufficient UAV verification ($Th_{uav}$) was configured to reflect a 10% global benchmark data proportion, and the data difference tolerance threshold ($\theta_{d}$) was set to 0.20. In the recommendation trust mechanism, the maximum recommendation level ($L_{max}$) was 2, and the recommender trust threshold ($\vartheta_{t}$) was 0.60. For the sequence extraction and trust computation stage, the lower and upper bounds for evidence classification were set to α=0.30 and γ=0.70, respectively; the time decay factor (β) was 0.50, and the exponential punishment intensity factor (σ) for untrustworthy behavior was 0.50.

For the newly proposed mechanisms in E-PTES-S, the regional consistency tolerance threshold ($\delta_{th}$) was set to 15.0 m (with a penalty coefficient $\lambda_{loc}=0.05$). For task timeliness, the optimal delay ($\tau_{opt}$) and maximum tolerable delay ($\tau_{max}$) were defined as 300 s and 900 s, respectively. The variance threshold ($\delta_{stb}$) for the CSS was strictly limited to 0.02, and its superlinear length reward exponent (λ) was set to 1.5. Through the dynamic scenario settings based on a real-world computing platform and trajectory data, this experiment comprehensively and objectively compared the performance of E-PTES-S against the PTES and Trust-SIoT baseline algorithms. The detailed parameter configurations for the experiment are summarized in Table 2.

### 5.2. Trust Evolution Analysis of Normal MDCs

**Figure 3:**
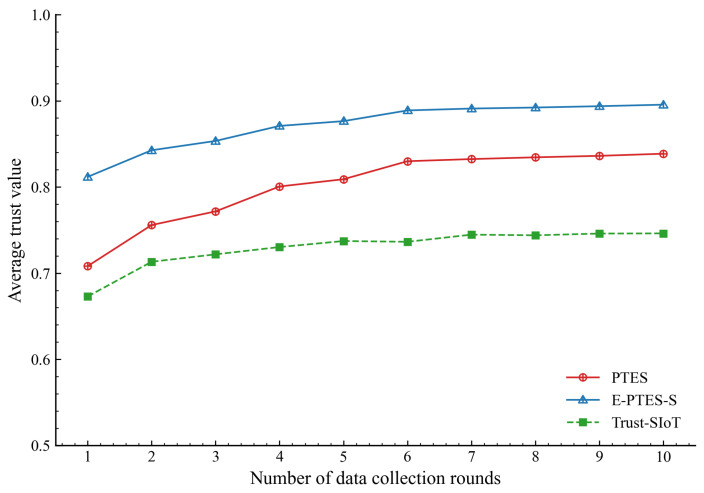
Average trust value evolution of normal MDCs.

**Figure 4:**
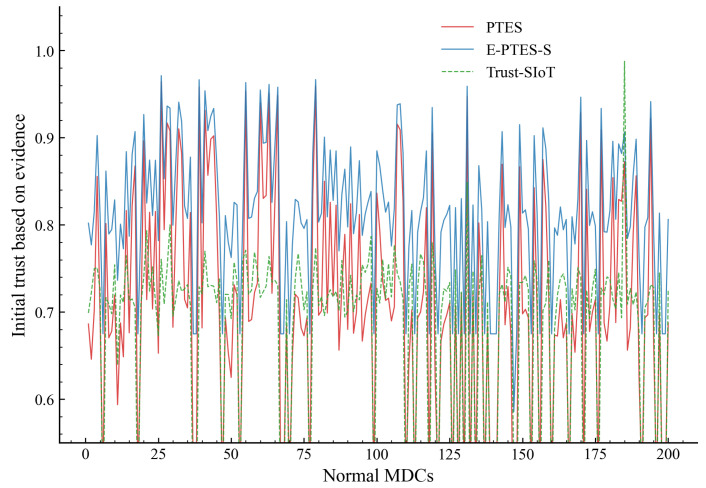
Initial evidence-based trust distribution of individual normal MDCs.

Figure 3 tracks the evaluation across 10 data collection rounds to map the shifting average trust scores of normal MDCs under the three tested schemes. In addition, as shown in Figure 4, the initial evidence-based trust distribution is mapped onto the nodes individually.

Referring to Figure 3, the average trust evaluation of E-PTES-S significantly exceeds that of both PTES and Trust-SIoT architectures. This phenomenon shows up immediately after the first set of experiments. Due to their prompt separation, it can be inferred from this that the multi-faceted integration approach reacts quickly enough with cooperation among healthy nodes; using $T_{RC}$ and $T_{TT}$ provides honest MDCs with an independent source of confirmation that they have been checked for correctness before release. The quick increase leads to an initial manifestation of all-around behavioural characteristics in the $T_{com}$ score.

Several rounds of operations lead to the overall convergence of all tested systems. After further observation on the slope of this graph, we find that E-PTES-S approaches equilibrium more quickly and settles to about 0.9 after some time. This accelerated convergence demonstrates the effectiveness of the mathematical design behind the $F_{CSS}$ reward mechanism. Submitting stable, low-variance data over several cycles triggers a superlinear reward through the CSS multiplier. The new architecture significantly reduces the time required for honest nodes to build a mature reputation. In practice, when a trustworthy score is raised earlier in a reliable device, it can eliminate the transaction threshold and increase the volume of legitimate data obtained by this system.

Isolating the granular data as shown in Figure 4 reveals a dense cluster of initial high-trust assignment by the E-PTES-S approach. This aggregation implies that, initially, scoring is more favourable to nodes with full spatiotemporal evidence. The evaluation engine shows high effectiveness in separating trustworthy nodes from the background noise even before deep historical sequences accumulate; it performs better than other schemes at the network’s entrance. While this section focuses on the stable convergence of trust values for normal nodes, the system’s capability to identify malicious entities will be specifically evaluated in the subsequent section.

### 5.3. Detection Rate Analysis of MDCs

The practicability of all trust assessment models depends on how effectively they can differentiate true legitimate data-collecting entities from highly deceptive malicious ones. To comprehensively evaluate this, we track the detection success rates of both normal and malicious MDCs over consecutive data collection rounds.

**Figure 5:**
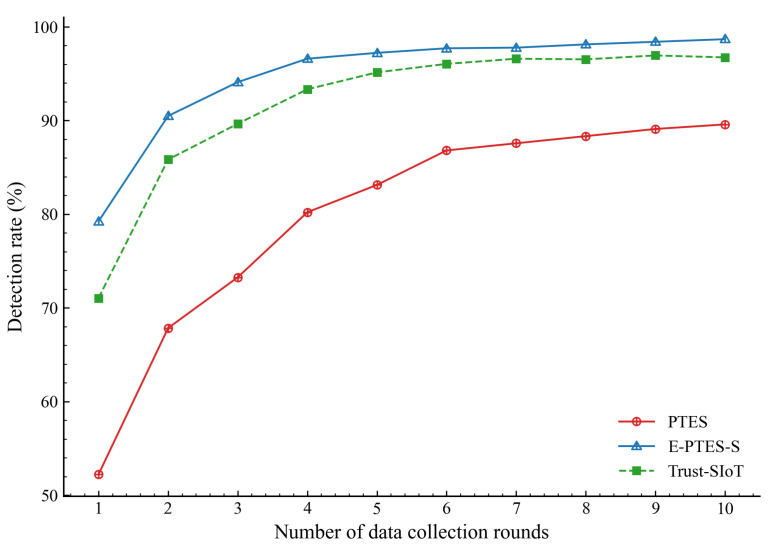
Evolutionary trend of the normal MDC detection rate.

As shown in Figure 5, it can be observed that E-PTES-S surpasses all other model types at a lower network startup detection rate. This rapid divergence indicates that the system accelerates the initial establishment of mutual trust. Since normally, the MDC does not send fake GPS position or sense data with intentional delays, it can generate a higher $T_{RC}$ and $T_{TT}$ score early on. Verified interactions lead to a complete trust evaluation of these users, surpassing the credibility threshold γ more quickly than systems without independent content verification. This quick evaluation effectively puts legitimate vehicles in the active-routing pool almost instantly.

**Figure 6:**
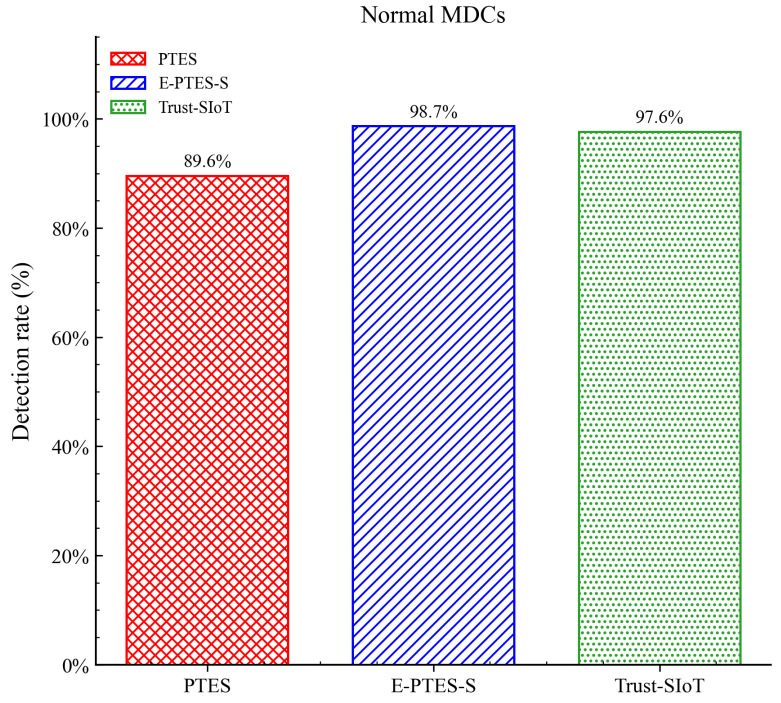
Comparison of the average detection rate of normal MDCs.

Figure 6 isolates the terminal data for comparison. The integrated evaluation of E-PTES-S reaches a detection rate of 98.7 per cent; it exceeds PTES by approximately 9 percentage points, and surpasses Trust-SIoT by about one percent. The nearly 98% recognition rate suggests that the stabilisation reward effectively eliminated spurious targets. In a live urban-sensing deployment, correctly identifying that almost all available honest collectors have been collected can maximise sensor coverage while reducing unnecessary communications.

**Figure 7:**
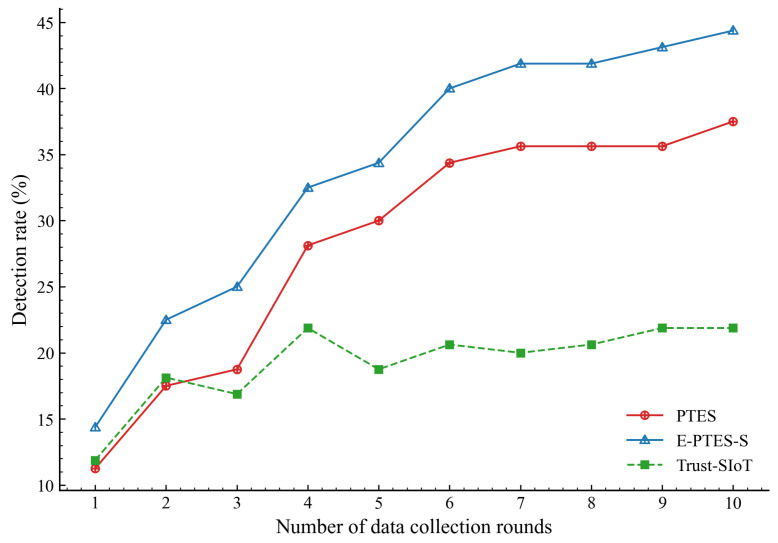
Evolutionary trend of the malicious MDC detection rate.

To further address the system’s defensive capabilities against insider threats, we evaluate the detection rates of malicious MDCs. As previously defined, these malicious entities execute concealed mixed attacks such as on–off attacks and data tampering, making them inherently difficult to identify. Figure 7 illustrates the evolutionary trend of identifying these attackers. It can be observed that E-PTES-S surpasses the baseline models and maintains a continuously widening advantage in capturing behavioral anomalies. While all models start with relatively low detection rates due to a lack of historical sequences, E-PTES-S significantly accelerates its identification process as data collection progresses.

**Figure 8:**
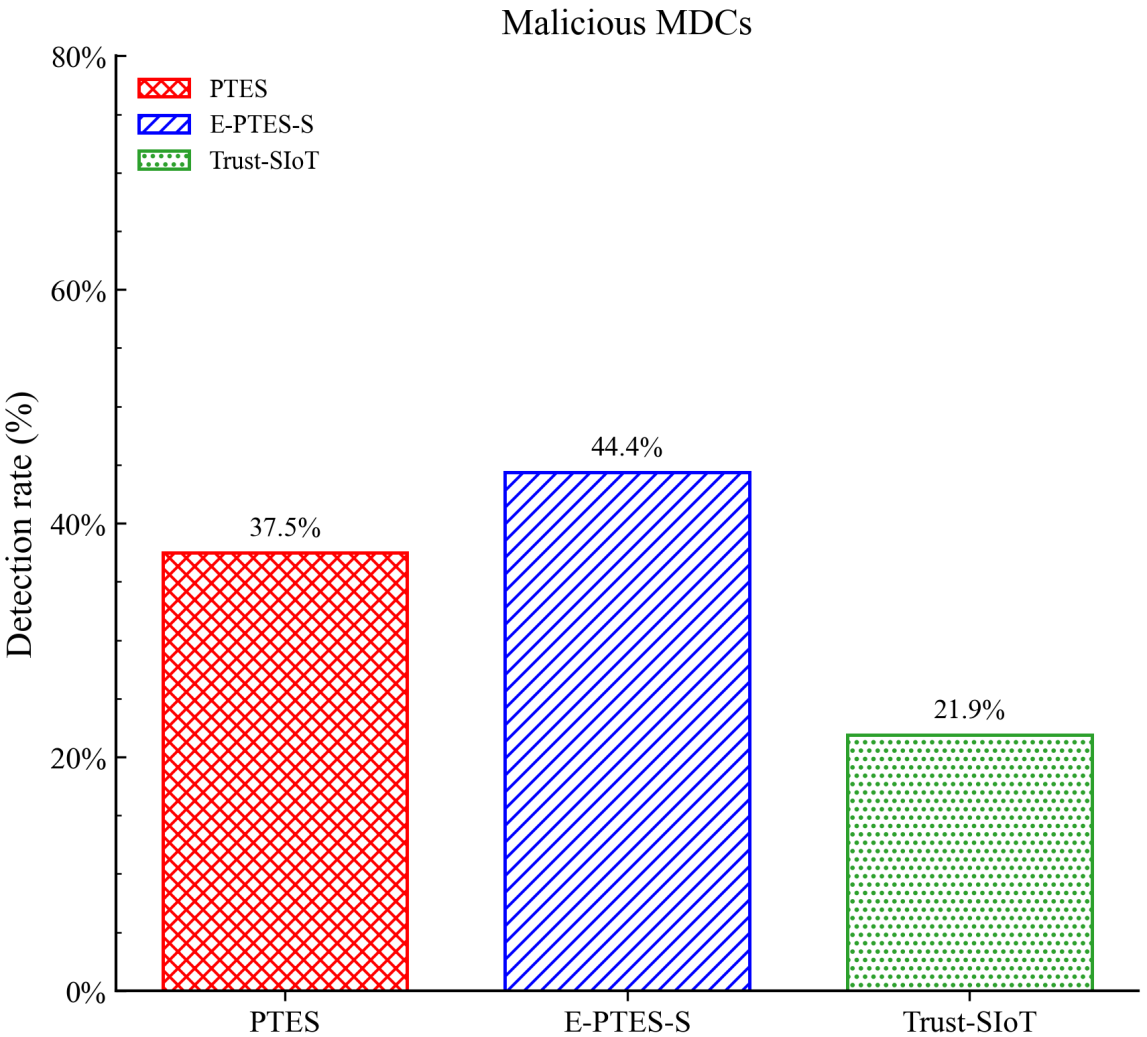
Comparison of the average detection rate of malicious MDCs.

As clearly shown in the terminal data comparison in Figure 8, the final malicious detection rate of E-PTES-S reaches 44.38%, exceeding the 37.50% of the baseline PTES by approximately 7% and outperforming the 21.88% of the Trust-SIoT framework. Although identifying highly deceptive on–off attackers is fundamentally challenging, this substantial relative improvement confirms the effectiveness of our proposed mechanisms. The variance-based $F_{CSS}$ mechanism effectively penalizes strategic volatility, successfully stripping away the attackers’ credible disguise. Furthermore, the inclusion of $T_{RC}$ and $T_{TT}$ independently catches physical-layer spatial spoofing and hoarding delays. Filtering out these deceptive entities efficiently reduces the risk of data poisoning in dynamic crowdsensing networks.

### 5.4. Data Collection Profit Analysis

Profit from data acquisition determines the economic feasibility of mobile crowdsensing systems. The system’s profit is proportional to the quantity and quality of received data packets. The normalised trust value of the MDC completely determines whether to accept it. We find that the accuracy of the trust architecture corresponds to profit-making in systems at different times. As a result, this approach enhances the accuracy and then improves economic benefit.

**Figure 9:**
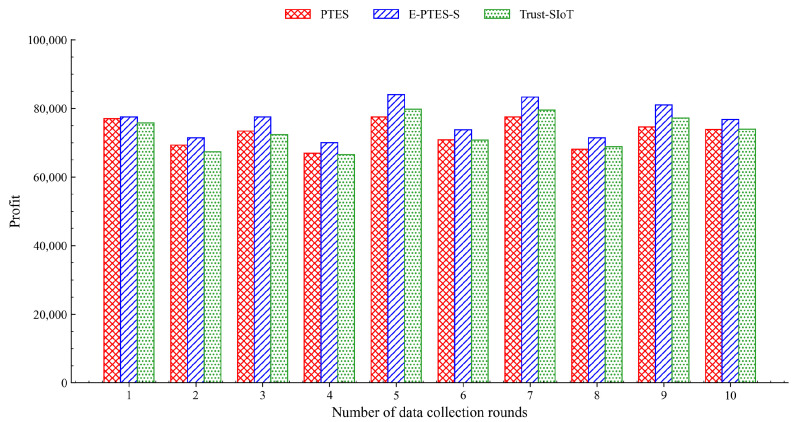
Comparison of per-round data collection profit.

**Figure 10:**
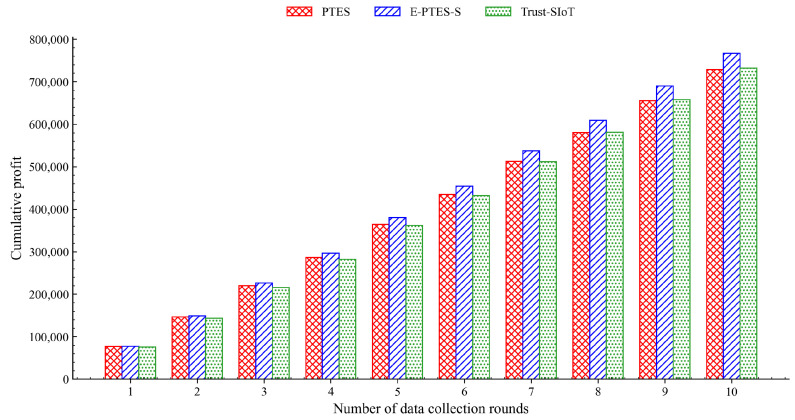
Comparison of cumulative profit.

Figure 9 shows the per-round data-collecting profit of all selected algorithms, and Figure 10 depicts their combined financial history.

As shown in Figure 9, the single-round profit of the E-PTES-S scheme is always higher than that of the PTES and Trust-SIoT schemes, because the E-PTES-S can quickly distinguish between faulty devices in high-purity environments, thus providing higher validity of transaction information and reducing the occurrence rate of financial frauds. Inherent intraround fluctuations of all three schemes are caused by changes in data volume collected over time when MDCs travel on different physical empirical curves, or move among varying sensor coverages.

As shown in Figure 10, the overall profit curve differences between the two schemes can also be seen clearly over time. Both curves show steady growth; however, the E-PTES-S scheme has a consistently high growth rate, and thus its cumulative gap increases over time. By the end of the experiment, the cumulative profit of the E-PTES-S scheme improves by approximately 4.8% compared to the PTES scheme. This represents the amplification of the positive impact in a real-world, massive crowdsourcing system. With a longer service life and wider participation, even a one-percentage-point increase in profit is worth a substantial amount of money.

### 5.5. Robustness Analysis Under Complex Wireless Conditions

To further evaluate the system’s capability to withstand physical-layer uncertainties, we conducted a robustness validation experiment focusing on the impact of environmental shadowing and multipath fading. In this scenario, we introduced a Gaussian noise model $X_{\sigma }∼N\left(0,\sigma_{env}^{2}+\sigma_{het}^{2}\right)$ into the distance estimation module, where the hardware heterogeneity variance $\sigma_{het}$ was fixed at 2.0 dB, and the environmental shadowing variance $\sigma_{env}$ was incrementally increased from 2 dB to 10 dB.

**Figure 11:**
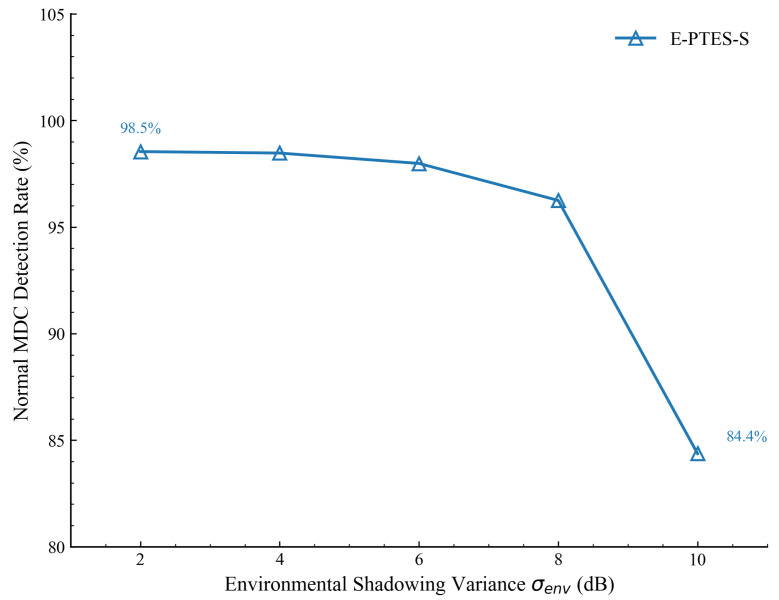
MDC detection rate under varying shadowing variance.

Figure 11 illustrates the MDC detection rate of the E-PTES-S scheme under these escalating noise levels. The results demonstrate its excellent robustness. In relatively stable environments where $\sigma_{env}\leq 6$ dB, the detection rate can be maintained at a level of around 98%. Even under severe environmental deterioration at 10 dB, although the detection rate of E-PTES-S decreases, it still successfully sustains a rate of 84.4%. This robust performance is consistent with the preceding theoretical error propagation analysis. Even under extreme noise interference where $\sigma_{env}$ reaches 10 dB, the multi-node collaborative mechanism can effectively suppress localized signal variance. This experiment validates the excellent robustness of the E-PTES-S scheme in maintaining reliable trust evaluation under physical-layer uncertainties.

### 5.6. Comprehensive Analysis and Discussion

The performance improvements of E-PTES-S are due to the combined effects of three innovations: Firstly, the multi-faceted trust fusion extends this model to incorporate a five-part system. $T_{RC}$ and $T_{TT}$ incorporate multiple pieces of independent information, thereby reducing biases in a single aspect. The second is the stable reward of the CSS mechanism. Combining the exponential decay penalty of credibility variance and superlinearly increasing lengths as rewards can accelerate the trust acquisition process for nodes with stable behavioural performance while also identifying certain malicious nodes. Finally, this enhanced evaluation accuracy for both benign and malicious behaviors translates into higher economic gains.

Despite the significant performance improvements achieved by E-PTES-S, we acknowledge that there are still several limitations and challenges to be addressed in real-world deployments. First, $T_{RC}$ relies too heavily on received signal strength indicator (RSSI) measurements. In dense urban canyons, the RSSI is highly susceptible to multipath fading, obstacle shadowing, and environmental noise, which could lead to false penalties for MDCs due to distance estimation errors. Furthermore, while the multi-node collaborative mechanism effectively mitigates random environmental noise, hardware heterogeneity across different MDC manufacturers still introduces static RSSI offsets. Future work could explore techniques such as relative RSSI variation tracking to mitigate the impact of hardware disparities on the trust evaluation process. Second, the proactive verification mechanism assumes the reliable and continuous availability of UAVs. In practice, UAV operations are often constrained by limited battery capacity, extreme weather conditions, and urban no-fly zones, potentially causing delays or blind spots in benchmark data acquisition. Finally, the current system relies on several predefined static empirical parameters, such as the CSS variance bound $\delta_{stb}$ and the classification thresholds α and γ. In highly dynamic and heterogeneous urban IoT environments, where network density and attacker strategies evolve rapidly, these fixed thresholds may lack optimal adaptability, impacting the model’s sensitivity when facing novel or unpredictable attack patterns.

Beyond these system-level constraints, it is also important to note that the performance trends presented in Figure 3, Figure 4, Figure 5, Figure 6, Figure 7, Figure 8, Figure 9 and Figure 10 are based on typical operational scenarios containing a moderate malicious node proportion of 10%. In extreme or highly hostile environments, these results may vary significantly. For instance, if a substantial number of malicious MDCs launch coordinated location spoofing attacks right from the network initialization phase by sending fake GPS positions early on, the initial average trust convergence shown previously in Figure 3 would experience a noticeable delay or a temporary dip. Although the $T_{RC}$ mechanism would still detect and penalize these malicious entities, the early network state would not appear as stable. Similarly, in a low-purity environment where the attacker ratio is relatively high, the massive rejection of anomalous data by the E-PTES-S filtering mechanisms would inevitably lead to a lower single-round and cumulative data collection profit than the levels depicted in Figure 9 and Figure 10. In these extreme cases, while E-PTES-S guarantees higher data security and validity compared to PTES, it trades off total collection volume, which impacts the overall economic yield of the crowdsensing system.

In summary, while there are practical challenges to overcome, through multi-dimensional trust fusion combined with the CSS mechanism, the E-PTES-S scheme improves the detection accuracy for both normal and malicious MDCs, enhances behavioral stability monitoring, and increases the comprehensive profitability of data acquisition in dynamic mobile crowdsensing networks. These identified limitations also provide a clear roadmap for our future optimizations.

## 6. Conclusions

This paper resolves the operational shortcomings of MDCs in a dynamic IoT sensor network environment. Distributed data collection is realised by presenting the E-PTES-S system. It integrates $T_{RC}$ and $T_{TT}$ in a five-dimensional baseline trust model structure, along with an expanded $F_{CSS}$ variation-based mechanism that assesses behavioural stability. We impose the above-specific structural constraints because traditional content-centred filters are highly vulnerable to spatiotemporal manipulation, including fake locations and delayed data poisoning. Simulations utilizing a real-world taxi trajectory dataset confirm the efficacy of this approach. The architecture accelerates trust convergence speed and improves the MDC detection rate, ultimately increasing cumulative data-collection profits. Moreover, robustness validations confirm the system’s excellent robustness against physical-layer uncertainties; even under severe environmental deterioration where $\sigma_{env}$ reaches 10 dB, the multi-node collaborative mechanism effectively suppresses localized signal variance to maintain reliable trust evaluation. Acknowledging current limitations regarding the susceptibility of RSSI measurements to environmental and hardware disparities, the inability to cope with extreme adversarial environments, and the lack of dynamic adaptability in static empirical parameters, future work will explore relative RSSI variation tracking and integrate lightweight machine learning algorithms, dynamically adjusting evaluation thresholds to further reduce computational overhead and enhance the system’s real-world applicability.

## Figures and Tables

**Table 1:** Main Symbols Used in E-PTES-S.

Symbol	Definition	Description or Constraint
$T_{PVT}$	Proactive verification trust	UAV benchmark comparison. Range: [0,1]
$T_{DIT}$	Direct interaction trust	Communication success/failure counts
$T_{RT}$	Recommendation trust	$J_{obj}\prod_{i=1}^{L_{rec}}R_{i,i+1}$; max tiers: 2
$T_{RC}$	Regional consistency trust	RSSI-based spatial verification. ∈[0,1]
$T_{TT}$	Task timeliness trust	Weighted average of $U_{time}\left(\tau_{x}\right)$
$T_{com}$	Comprehensive trust value	Adaptive fusion of five dimensions
α,γ	Classification thresholds	[0,α): untrustworthy; [α,γ): uncertain; [γ,1]: credible
$F_{CCS}$	Credible sequence influence	Time-decayed positive contribution
$F_{CUS}$	Untrustworthy sequence influence	Exponential penalty for malicious behavior
$F_{CUCS}$	Uncertain sequence influence	Proportional, no penalty
$F_{CSS}$	Stability sequence influence	${\left(L_{j}^{CSS}\right)}^{\lambda }\cdot e^{-\mathrm{Var}(S_{j}^{CSS})}$
$Tru_{i}$	Final normalized trust	Output trust of MDC *i*; ∈[0,1]
β	Time decay factor	0<β<1; favors recent evidence
$\delta_{stb}$	Stability threshold	Variance bound for CSS; default 0.02
λ	Length reward exponent	CSS superlinear reward; default 1.5
$\theta_{d}$	Data difference threshold	Tolerance for proactive verification
$\delta_{th}$	Spatial deviation tolerance	Max allowed $|d_{est}-d_{claim}|$
$\tau_{opt},\tau_{max}$	Timeliness bounds	Optimal and maximum upload delay
$w_{pri}$	Data priority weight	Importance weight per data packet
*Z*	Sliding window size	Retained evidence records; default 10

**Table 2:** Experimental Parameter Settings.

Parameter	Symbol	Value
Number of MDCs	*N*	1600
Sliding window size	*Z*	10
UAV data sufficiency threshold	$Th_{uav}$	10% global proportion
Penalty intensity factor	σ	0.50
Data exchange trust threshold	-	0.50
Recommender trust threshold	$\vartheta_{t}$	0.60
CSS variance threshold	$\delta_{stb}$	0.02
Minimum CSS length	$L_{min}^{CSS}$	3
Number of sensing devices	*K*	500
Data difference threshold	$\theta_{d}$	0.20
Maximum recommendation tiers	$L_{max}$	2
Number of data collection rounds	-	10
Evidence classification thresholds	α/γ	0.30/0.70
Time decay factor	β	0.50
CSS length reward exponent	λ	1.5
Proportion of malicious attackers	-	10% × *N*

## Data Availability

Publicly available datasets were analyzed in this study. The T-Drive taxi trajectory dataset can be found at the official Microsoft Research repository: https://www.microsoft.com/en-us/research/publication/t-drive-trajectory-data-sample/ (accessed on 1 April 2026), and is described in detail in reference [37].
